# Potential of Tryptamine Derivatives as Multi-Target Directed Ligands for Alzheimer’s Disease: AChE, MAO-B, and COX-2 as Molecular Targets

**DOI:** 10.3390/molecules29020490

**Published:** 2024-01-19

**Authors:** Saira Asghar, Nousheen Mushtaq, Ahsaan Ahmed, Laila Anwar, Rabya Munawar, Shamim Akhtar

**Affiliations:** 1 Department of Pharmaceutical Chemistry, Faculty of Pharmacy, Hamdard University, Karachi 74600, Pakistan; dean.fop@hamdard.edu.pk; 2 Department of Pharmaceutical Chemistry, Faculty of Pharmacy and Pharmaceutical Sciences, University of Karachi, Karachi 75270, Pakistan; nmushtaq@uok.edu.pk; 3 Institute of Pharmaceutical Sciences, Jinnah Sindh Medical University, Karachi 75510, Pakistan; ahsaan.ahmed@jsmu.edu.pk; 4 Department of Pharmacology, Faculty of Pharmacy, Hamdard University, Karachi 74600, Pakistan; laila.anwer@hamdard.edu.pk; 5 Department of Pharmaceutical Chemistry, Dow College of Pharmacy, Dow University of Health Sciences, Karachi 74200, Pakistan; rabya.munawar@duhs.edu.pk

**Keywords:** tryptamine derivatives, Alzheimer’s disease, multi-target-directed ligands (MTDLs), acetylcholinesterase (AChE) inhibitors, monoamine oxidase (MAO-B) inhibitors, cyclooxygenase (COX-2) inhibitors, enzyme inhibitors

## Abstract

Extensive research has been dedicated to develop compounds that can target multiple aspects of Alzheimer’s disease (AD) treatment due to a growing understanding of AD’s complex multifaceted nature and various interconnected pathological pathways. In the present study, a series of biological assays were performed to evaluate the potential of the tryptamine analogues synthesized earlier in our lab as multi-target-directed ligands (MTDLs) for AD. To assess the inhibitory effects of the compounds, various in vitro assays were employed. Three compounds, **SR42**, **SR25**, and **SR10,** displayed significant AChE inhibitory activity, with IC_50_ values of 0.70 µM, 0.17 µM, and 1.00 µM, respectively. These values superseded the standard drug donepezil (1.96 µM). In the MAO-B inhibition assay, **SR42** (IC_50_ = 43.21 µM) demonstrated superior inhibitory effects as compared to tryptamine and other derivatives. Moreover, **SR22** (84.08%), **SR24** (79.30%), and **SR42** (75.16%) exhibited notable percent inhibition against the COX-2 enzyme at a tested concentration of 100 µM. To gain insights into their binding mode and to validate the biological results, molecular docking studies were conducted. Overall, the results suggest that **SR42**, a 4,5 nitro-benzoyl derivative of tryptamine, exhibited significant potential as a MTDL and warrants further investigation for the development of anti-Alzheimer agents.

## 1. Introduction

Alzheimer’s disease (AD) remains a complex and multifaceted neurodegenerative condition associated with aging and is now among the top eight global health challenges. According to the Alzheimer’s Association, AD affects approximately 5.8 million people in the United States, with about 5.6 million of them being aged 65 and older, and the remaining 0.2 million under the age of 65 [1]. AD is characterized by a gradual decline in memory and cognitive functions. Globally, more than 47 million individuals currently suffer from dementia, and the overall cost of dementia care worldwide is estimated to be around USD 818 billion. Regrettably, this number is projected to surge to 131.5 million by the year 2050 [2,3]. This disease progresses from mild to severe, and notably, neurodegenerative changes in the brain can be initiated up to 20 years before observable symptoms manifest. AD is the most common cause of dementia among older adults and is characterized by the formation of neurofibrillary tangles within and Aβ plaques outside the cells, which are significant pathological hallmarks. It is considered a progressive and chronic condition, affecting various cognitive functions, including memory, learning, orientation, thinking, language, comprehension, and judgment. It has also been noted that neurodegenerative diseases involve multiple pathways that are interconnected, either directly or indirectly. Consequently, AD is now recognized as a multifactorial condition, in which multiple pathways contribute to its progression. Multiple molecular mechanisms underlie AD pathogenesis, including reduced acetylcholine levels in the brain, abnormal deposition of amyloid beta (Aβ)-peptide leading to plaque formation, hyperphosphorylation of tau (τ) protein resulting in neurofibrillary tangles, oxidative stress, inflammatory responses, synaptic loss, high levels of N-methyl-D-aspartate (NMDA), β-secretase activity, and genetic mutations [4,5,6]. From a biochemistry standpoint, neurodegenerative diseases have shared pathological mechanisms, including protein misfolding and accumulation, changes in neurotransmitter levels (e.g., acetylcholine and dopamine), imbalances in metal ion regulation, malfunctioning of mitochondria, oxidative stress, and neuroinflammation [7,8].

Five drugs have received FDA approval for AD treatment, including the N-methyl-D-aspartate (NMDA) receptor antagonist memantine and acetylcholinesterase inhibitors (AChEIs) such as tacrine, rivastigmine, donepezil, and galantamine. Tacrine, the initial potent and clinically effective acetylcholinesterase (AChE) inhibitor, secured FDA approval in 1993 for Alzheimer’s disease (AD) treatment. Unfortunately, owing to concerns about its hepatotoxicity, the drug was subsequently withdrawn from the pharmaceutical market in 1998 [9]. These medications can only provide limited improvement in memory and cognitive function. They do not halt or reverse AD progression. Early in the disease progression, the cholinergic systems are impacted, leading to a reduction in acetylcholine levels within neurons. This disruption also affects the enzymes responsible for both the production and breakdown of acetylcholine, a critical neurotransmitter for communication among nerve cells. As a result, the primary goal of AChEIs is to elevate acetylcholine levels by inhibiting acetylcholinesterase (AChE), the enzyme responsible for acetylcholine’s degradation [10,11,12]. Currently, treatment using anti-Aβ monoclonal antibodies such as aducanumab and lecanemab has demonstrated notable success in animal models of AD, leading to enhancements in cognitive functions and a reduction in brain pathology. Recently, both aducanumab and lecanemab have obtained FDA approval for addressing AD with mild cognitive impairment. Aducanumab has exhibited a distinct therapeutic impact, effectively clearing Aβ from the brains of mice. Similarly, lecanemab has demonstrated the ability to diminish pathogenic Aβ, prevent Aβ deposition, and selectively reduce Aβ protofibrils in both the brain and cerebrospinal fluid (CSF) of AD animal models [13,14]. Moreover, the continuous production and aggregation of Aβ peptides, particularly Aβ (1–40) and Aβ (1–42), contribute to neurodegeneration and eventual neuronal dysfunction. Amyloid-β (Aβ), a prominent pathological feature in AD, is produced through a stepwise proteolytic mechanism that commences with the cleavage of the amyloid precursor protein (APP). This cleavage occurs in the presence of enzymes such as β-site APP-cleaving enzyme 1 (BACE1) and γ-secretase. This process leads to the creation of Aβ, with Aβ42 being its principal constituent. As a result, inhibiting Aβ production and accumulation in the brain has been identified as a potential therapeutic target. Additionally, the progressive aggregation of Aβ is closely linked to oxidative stress, a significant factor in AD pathogenesis. Oxidative damage, marked by nitration, reactive carbonyls, lipid peroxidation, and nucleic acid oxidation, is elevated in vulnerable neurons in AD. Protecting neuronal cells from oxidative damage could potentially slow down the progression of AD [11,15,16,17,18]. Monoamine oxidase A (MAO-A) is responsible for the deamination of specific neurotransmitters like serotonin, noradrenaline, and dopamine [19]. In contrast, MAO B primarily degrades compounds such as phenylethylamine and benzylamine. Both isoforms are linked to the pathology of AD and are potential targets for intervention. MAO-A is associated with toxicity and neuronal cell death, whereas MAO-B is connected with the processing of Aβ peptide. Additionally, other processes, including oxidative stress, neuroinflammation, disruptions in calcium and metal ion regulation, excitotoxicity, and mitochondrial damage, are also considered significant in the development of therapeutic drugs for AD [20,21,22].

**Figure 1:**
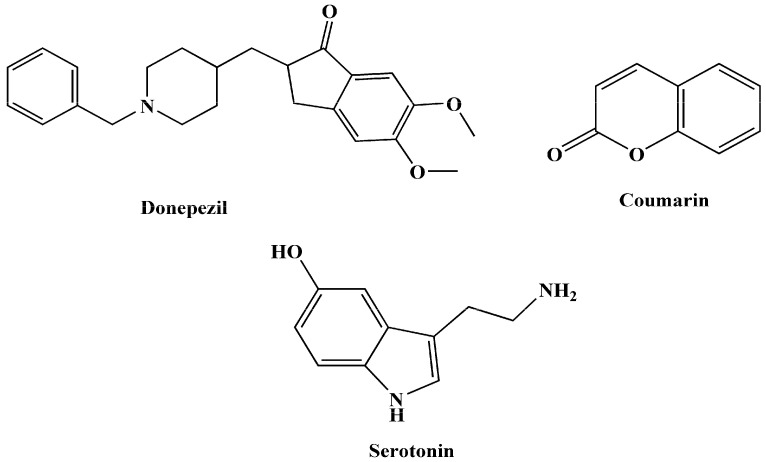
Some promising structures to create innovative MTDLs for AD.

Given the intricate pathogenesis and progression of AD, targeting a single pathway may prove insufficient. As a result, a multi-target approach, following the multi-target-directed ligands (MTDLs) paradigm, has emerged as a more efficient therapeutic strategy. In modern medicinal chemistry, this approach involves designing multifunctional compounds that can modulate various receptors and enzymes, aiming to address the multifactorial causes of AD. By combining effective pharmacophoric groups within a single molecule, MTDLs can provide superior therapeutic outcomes compared to single-target compounds with high affinity for one or a few targets [23,24,25,26,27]. Certain molecular scaffolds serve as excellent starting points for the efficient development of new lead MTDLs to treat AD. This subset of molecules has been utilized in the design and synthesis of hybrid therapeutic compounds for AD. Within this context, specific compounds (Figure 1) can be regarded as privileged initial structures, providing a foundation for the creation of novel MTDL leads in the context of AD [28,29].

Tryptamine, a monoamine alkaloid containing an indole ring, is of particular interest in AD research. It is derived from tryptophan through decarboxylation and is present in trace amounts in the brains of mammals, where it serves as a neuromodulator, neurotransmitter, and, in certain cases, a hallucinogenic agent. Tryptamine derivatives, achieved through synthetic alterations, have demonstrated dynamic effects on human mental and physical conditions. Melatonin, a derivative of tryptamine, has shown promise in halting tau protein hyperphosphorylation and protecting against Aβ peptide toxicity in AD [30,31,32].

Researchers have also designed N-salicyloyl tryptamine analogs as MTDLs for AD and Parkinsonism treatment. These analogs involve the conjugation of salicylic acid with tryptamine, resulting in different compounds with potential as multi-functional agents for neuroinflammatory-linked neurodegenerative disorders [33]. In addition, hybrid compounds, combining melatonin with other substances such as tacrine, have been designed as MTDLs for AD therapy, reducing Aβ peptide toxicity, oxidative stress, and enhancing cholinergic neurotransmission. Molecular docking studies have revealed interactions with key enzymes involved in AD pathogenesis [34]. Idalopirdine, a hybrid of a tryptamine moiety and benzylamine, is reported as the most advanced anti-AD hybrid compound that succeeded in reaching phase-3 clinical studies in AD patients [28].

**Figure 2:**
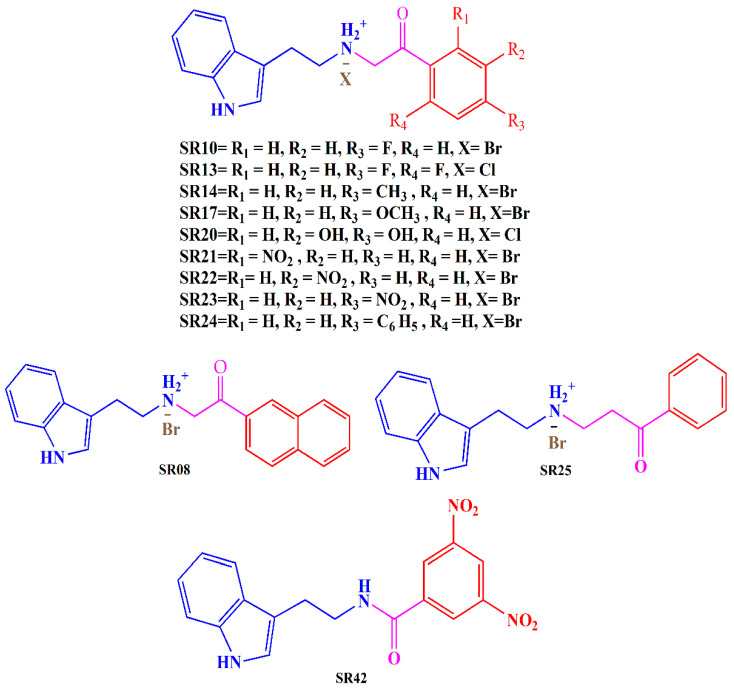
Synthesized tryptamine derivatives.

In light of these findings and our ongoing commitment to developing potent MTDLs for AD, we have evaluated the biological activity of previously designed and synthesized tryptamine-based derivatives [35]. Our goal was to investigate the inhibitory potential of these compounds against multiple targets, including AChE, MAO-B, and COX-2. Derivatives were synthesized by reacting tryptamine nuclei with various phenacyl, naphthyl, and benzoyl halides (Cl or Br). The structures of the tryptamine derivatives were comprised of three sections: (1) a blue part that represents the parent nucleus of tryptamine, (2) a purple portion depicting the connecting chain, and (3) a red area showing the aromatic lipophilic region (Figure 2). Docking studies have been employed to gain insights into the binding mode of the compounds and to support the in vitro results.

## 2. Result and Discussion

### 2.1. In Vitro Enzyme Inhibition Investigation

The AChE inhibition potential of twelve synthesized tryptamine derivatives was investigated through the Ellman method, with few modifications. Donepezil and galantamine were used as standards. The IC_50_ values of all the compounds are summarized in Table 1. Derivatives demonstrated IC_50_ values ranging from 0.176 to 87.73 µM. **SR25**, **SR42**, and **SR10** possessed better AChE inhibition activity than donepezil. Compounds revealed the inhibitory potential in order of potency as **SR25** > **SR42** > **SR10** > **Donepezil** > **SR08** > **SR24** > **SR23** > **SR17** > **SR13** > **Galantamine** > **SR20** > **SR22** > **SR14** > **SR21** > **tryptamine**.

In this investigation, the primary objective was to evaluate the suitability of certain tryptamine derivatives with promising in vitro activity as potential lead compounds for AD.

All the synthesized derivatives demonstrated better IC_50_ values than the parent, indicating the impact of substitution in the tryptamine nucleus. Among the series, **SR25**, a propiophenone derivative, exhibited the most promising AChE inhibitory potential (IC_50_ = 0.176 ± 0.02 µM), which was eleven-fold higher than donepezil (IC_50_ = 1.96 ± 0.41 µM). **SR42**, a *meta*-di-nitro benzoyl derivative, presented the second-best AChE inhibitory activity (IC_50_ = 0.70 ± 0.21 µM), with three times better activity than standard. Replacement of di-nitro to a single *para*-nitro group in **SR23** dramatically reduced the activity of the molecule, with an IC_50_ value of 7.33 ± 0.12 µM, which further dropped when nitro was moved to the *meta* position in **SR22** (IC_50_ = 54.88 ± 0.19 µM). Shifting of nitro at the ortho position in **SR21** further weakened the enzyme inhibitory power, with an IC_50_ value of 87.73 ± 0.16 µM. The nitro group in the structure is known for its toxicity, and it is often perceived as toxicophore, for mutagenicity and genotoxicity. The nitro group stands out as a distinctive functional group in medicinal chemistry due to its robust electron-withdrawing and highly polar nature. The general belief that the nitro group negatively affects beneficial biological activity has been quite common. However, this is not true, as a number of drugs that are actively used as antihypertensive, antianginal, antiarrhythmic, anti-Alzheimer, and anti-Parkinson’s agents contain a nitro group. Recently, there has been a renewed interest in nitro drugs, exemplified by the approval of fexinidazole for treating human African trypanosomiasis (HAT), a neglected tropical disease [36,37,38].

**SR10** (*para*-fluoro phenacyl tryptamine derivative) showed good performance, with two-fold better inhibition (IC_50_ = 1.0 ± 0.080 µM) than donepezil, whereas the addition of one more fluoro group at the *ortho* position in **SR13** remarkably reduced the inhibitory potential (IC_50_ = 9.68 ± 0.16 µM) of the molecule. **SR20** (*meta*, *para*-dihydroxy phenacyl) is another disubstituted derivative with polar groups, but here, activity dropped significantly, with an IC_50_ value of 24.97 ± 0.05 against the AChE enzyme.

Though **SR08** and **SR24** produced better inhibitory potential than galantamine among the series, they performed better than the number of compounds with IC_50_ values of 4.36 ± 0.12 µM and 6.70 ± 0.08 µM, respectively. Both molecules have large structures, and the replacement of *para*-phenyl benzene rings (**SR24**) with a naphthyl ring (**SR08)** enhanced the inhibition potential.

The *para*-methyl phenacyl derivative (**SR14**) displayed an IC_50_ value of 68.71 ± 0.24 µM, and the replacement of methyl with the methoxy group in **SR17** not only induced approximately eight times better inhibitory potential (IC_50_ = 8.23 ± 0.12 µM) than **SR14** but also superseded the activity of galantamine.

The MAO-B enzyme inhibition activity was assessed using the modified Amplex Red monoamine oxidase assay kit [39]. The results are presented in Table 1. The synthesized tryptamine derivatives exhibited varying degrees of activity against MAO-B, with IC_50_ values ranging from 43.21 to 244.2 µM. In terms of MAO-B inhibition, the order of potency from highest to lowest was as follows: **pargyline** > **SR42** > **SR13** > **SR22** > **SR25** > **SR23** > **SR14** > **SR24** > **SR21** > **SR17** > **SR10** > **SR20** > **SR08** > **tryptamine**. All the synthesized tryptamine analogs exhibited greater activity than tryptamine itself but lower inhibition potential than the standard pargyline (IC_50_ value of 23.37 ± 0.32 µM).

Notably, successful AChE inhibitors **SR42** and **SR25** again exhibited good MAO-B inhibition within the series, with IC_50_ values of 43.21 ± 0.46 µM and 85.1 ± 0.26 µM, respectively.

Substitution of flouro at the *ortho* and *para* position produced a substantial effect on the MAO-B inhibition, as displayed by **SR13**, with an IC_50_ value of 50.64 ± 0.64 µM, whereas removal of the *ortho*-flouro group in **SR10** reduced the inhibition potential more than four-fold (IC_50_ = 216.10 ± 0.29 µM). Interestingly, **SR13** exhibited weaker AChE inhibition than **SR10**, but against MAO-B, it superseded **SR10**. The presence of *meta*, *para*-dihydroxy groups in **SR20** further dropped the activity (IC_50_ = 222.13 ± 0.32 µM).

Among the three nitro phenacyl analogs, nitro at the *meta* position in **SR22** demonstrated superior MAO-B inhibition, with an IC_50_ value of 56.46 ± 0.4 µM, compared to nitro at the *para* (**SR23**) and *ortho* (**SR21**) position, with IC_50_ values of 95.10 ± 0.43 µM and 142.13 ± 0.32 µM, respectively.

The para-methyl group in **SR14** did not succeed in producing a good impact on enzyme inhibition and displayed an IC_50_ value of 112.20 ± 0.29 µM. Contrary to AChE inhibitory activity, the replacement of methyl with methoxy in **SR17** reduced the inhibition activity, yielding an IC_50_ value of 150.10 ± 0.26 µM.

Bulky lipophilic substitution at the terminal position in **SR08** (naphthyl) and **SR24** (*para*-phenylbenzene) did not improve the enzyme inhibition and exhibited IC_50_ values of 244.20 µM ± 1 and 133.21 ± 0.46 µM, respectively.

The in vitro COX-2 inhibition was assessed using a fluorometric COX-2 inhibition assay kit. The results were tabulated in Table 1. The assay employed a fluorescence-based technique to detect the immediate compound (prostaglandin) formed as the end product, allowing for accurate measurement of COX-2 inhibition. Structure–activity relationships (SAR) revealed that the position, type, and number of substituents, along with the connecting moiety, played a crucial role in COX-2 inhibition. Molecules followed the order of COX-2 inhibition as: **celecoxib** > **indomethacin** > **SR22** > **SR24** > **SR42** > **SR13** > **SR10** > **SR17** > **SR25** > **SR08** > **SR23** > **SR20** > **SR14** > **tryptamine** > **SR21**.

Both positive controls, celecoxib and indomethacin, exhibited significant percentage inhibition values of 87.86 ± 0.63% and 87.03 ± 0.57%, respectively. Tryptamine displayed a moderate inhibition of 57.33 ± 4.96%. The COX-2 inhibition potential of the synthesized derivatives ranged from 34.43% to 84.08%.

Among synthesized compounds, the most potent inhibitor was **SR22**, a *meta*-nitro phenacyl derivative, with a notable inhibition activity of 84.08 ± 4.53%. The position of the nitro group made a striking impact on the power of COX-2 inhibition, as its shifting to the *para* position in **SR23** reduced the activity (69.40 ± 3.11%). This enzyme-inhibitory potential diminished to the lowest point among the synthesized derivatives when the nitro group was moved to the *ortho* position in **SR21** (34.43 ± 3.34%). *Para*-phenyl benzene in **SR24** displayed substantial inhibition at 79.30 ± 2.02%. Replacing the terminal aromatic rings with naphthyl in **SR08** lowered the inhibition power of the enzyme (72.33 ± 1.6%). **SR42** and **SR25** displayed significant inhibition (75.16 ± 2.30% and 72.43 ± 2.36%) against the enzyme.

**SR13**, an *ortho*, *para*-di-fluoro phenacyl derivative, exhibited 73.83 ± 2.37% inhibition. Removing the *ortho*-flouro group in **SR10** did not make a big difference in the inhibition of the enzyme (72.9 ± 2.42%). The *meta*, *para*-dihydroxy phenacyl derivative (**SR20**) demonstrated a good inhibition of 68.66 ± 3.86% against the COX-2 enzyme.

The *para*-methyl phenacyl ring in **SR14** yielded a moderate inhibition of 65.26 ± 2.65%, and replacing methyl with methoxy in **SR17** enhanced the inhibitory potential to 72.83 ± 2.54%.

These findings highlight the potential of the synthesized compounds to mitigate COX-2-related neuroinflammation, in addition to their inhibitory effects on AChE and MAO-B, positioning them as promising candidates for the treatment of AD.

### 2.2. Molecular Docking against Acetylcholinestrase Enzyme

The approach involved utilizing the human recombinant acetylcholinesterase (hAChE) target protein, identified by its PDB ID: 4EY7, and employing molecular docking techniques to understand how these derivatives interact with the target. 4EY7 is a Homo sapiens protein, with the donepezil as a co-crystalized ligand. Notably, donepezil exhibits a disease-modifying effect, offering neuroprotection, and it is currently used as an effective drug for AD. The selection of this protein was further supported by the quality of the model, characterized by more than 90% of residues residing in the most favored region. This underscores the appropriateness and reliability of the chosen model for the intended research or analysis [40,41].

**Figure 3:**
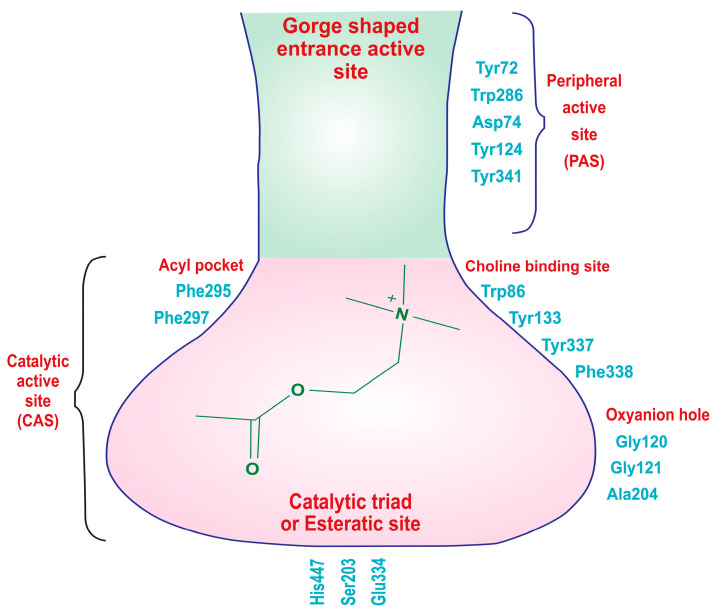
Two distinct types of binding sites of the acetylcholinesterase (AChE) enzyme: the catalytic active site (CAS) and the peripheral active sites (PAS).

The X-ray crystallographic structure of hAChE reveals two distinct active sites: the catalytic active site (CAS), located at the bottom of a narrow gorge, and the peripheral active site (PAS) near the gorge’s entrance (Figure 3). The CAS is primarily composed of specific amino acid residues, including Trp86, Gly122, Ala204, Phe295, Phe297, Ser203, Glu334, and His447, with the catalytic triad consisting of Ser203, Glu334, and His447, which are crucial for choline hydrolysis. Additionally, amino acids like Trp86, Tyr133, Tyr337, and Phe338 engage in cation–π interactions with the quaternary ammonium group of acetylcholine, aiding in substrate recognition. The acyl pocket, formed by Phe295 and Phe297, plays a role in substrate selectivity, and Gly121, Gly122, and Ala204 are part of the oxyanion hole, facilitating hydrogen bond formation in the transition state of acetylcholine hydrolysis. The hydrophobic active site interacts with the alkyl portion of acetylcholine. On the other hand, the PAS, positioned near the gorge entrance, involves amino acid residues like Tyr72, Asp74, Trp286, Tyr124, Tyr337, and Tyr341. These residues are responsible for stabilizing substrate binding, contributing to the overall efficiency of the enzyme. In summary, hAChE’s structural features enable it to efficiently regulate neurotransmission by breaking down acetylcholine, with the CAS handling enzymatic activity and the PAS aiding in substrate recognition and stabilization [42,43,44,45].

The endogenous ligand, acetylcholine (ACh), consists of three distinct components: an acetate group, an ethylene chain, and a choline moiety. Each of these fragments possesses specific structural requirements to bind with the acetylcholinesterase (AChE) enzyme. The choline moiety, specifically the charged nitrogen, binds to the anionic site, and the acetate group interacts with the esteratic site in the active binding region. This is the site where the primary hydrolysis reaction occurs, leading to the release of the choline metabolite. Meanwhile, the ethylene bridge interacts with the acyl site of the enzyme [46,47].

**Figure 4:**
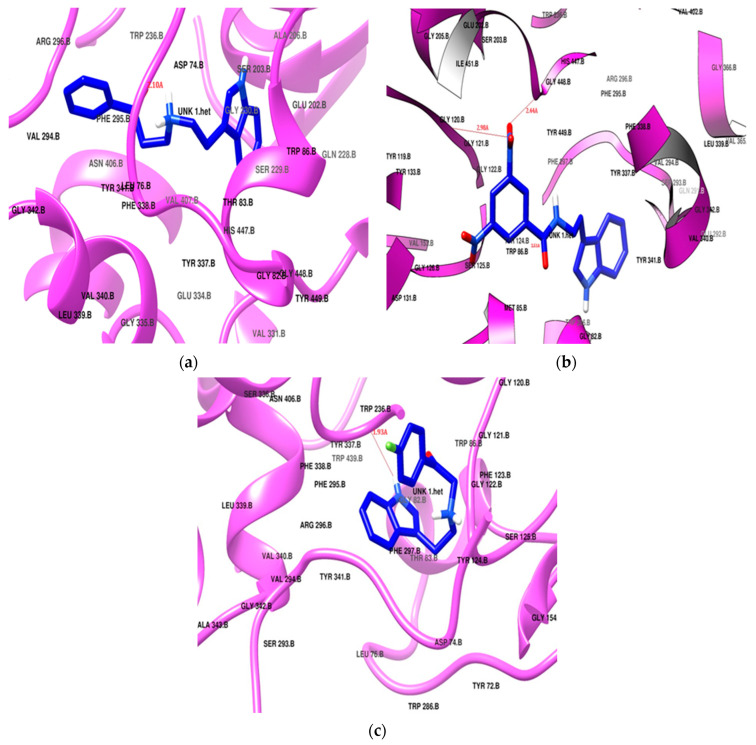
Potential docking position of (**a**) SR25, (**b**) SR42, (**c**) SR10. All ligands are presented in blue stick model (PDB: 4EY7).

Donepezil exhibited the highest docking score of −11.2 kcal/mol, positioning itself in both of the active sites, the PAS and CAS, of the enzyme cavity via hydrogen bonding with Tyr124 and Phe295. Donepezil was surrounded by Tyr72, Tyr124, Trp286, and Tyr341 in the PAS, and it interacted with the choline-binding site of the CAS through Trp86, Tyr337, Phe338, His447, and Gly448 as a hydrophobic lining. Two phenylalanine residues (Phe29 and Phe297) had hydrophobic interactions in the acyl pocket of the enzyme. Gly120 and Gly121 from the oxyanion hole were found to engage in hydrophobic bonding with the donepezil. **SR25** and **SR42** also showed engagement of the same active site amino acid residues in both the PAS and the CAS. The preferred orientation of these derivatives after the docking is presented in Figure 4. All the docking results are presented in Table 2.

The nitro phenacyl derivative **SR42** achieved a docking score of −10.4 kcal/mol, displaying a dual-site binding behavior: the amine, linker, and substituted aromatic ring interacted with the CAS region, and the ethyl indole of the tryptamine settled in the PAS. The carbonyl of the linker established a hydrogen bond with Tyr124, securely anchoring **SR42** within the PAS of the AChE enzyme (Figure 4b). One nitro group of the terminal aromatic ring significantly produced two hydrogen bonds with Gly121 and His447 in the CAS. The involvement of the nitro and carbonyl groups in the hydrogen bonding with the PAS and CAS gave stronger interactions with the enzyme and might be the reason for the better in vitro AChE inhibition activity.

The propiophenone derivative **SR25** flipped as it entered into the enzyme and, unlike the **SR42** ethyl indole, moved deeply into the CAS; the rest of the molecule settled in the PAS region. **SR25** (docking score −9.7 kcal/mol) successfully established a hydrogen bonding between alkylamine and Tyr124 (Figure 4a). The increased length of the linker allowed for the penetration of the indole ring deeper into the hydrophobic region at the bottom of the CAS and made the molecule the most successful enzyme blocker, which was also validated by the in vitro enzyme inhibition results. Both **SR42** and **SR24** exhibited dual-site inhibition of the AChE enzyme, and it is reported in the literature that dual binding site inhibitors are helpful to reduce beta amyloid fibril formation [48].

**SR10** (Figure 4c), with a docking score of −8.7 kcal/mol, exclusively positioned itself within the CAS region of the enzyme through hydrophobic interactions. Notably, Tyr337 created hydrogen bonds with the amine of indole, stabilizing its stay in the cavity. Both the ends of the molecule bent towards each other like the galantamine standard, but **SR10** showed better in vitro results, which may be linked with more hydrophobic interacting residues as compared to the galantamine standard.

### 2.3. Molecular Docking against Monoamine Oxidase-B Enzyme

**Figure 5:**
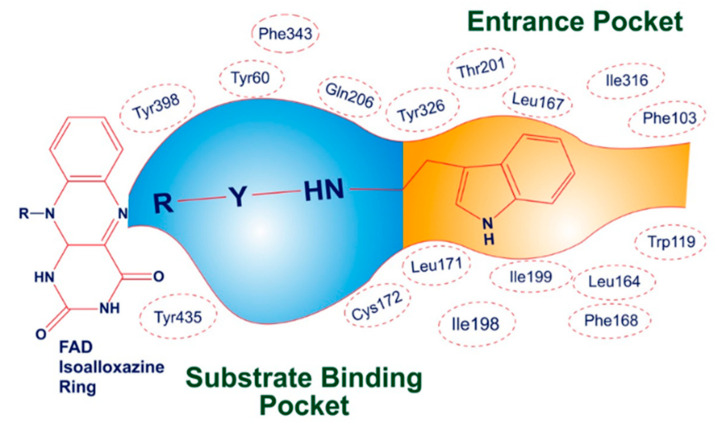
Illustrative depiction of how **SR42** interacted within the active site of the MAO-B enzyme.

The three-dimensional crystallographic structure reveals that MAO-B is found in a dimeric form. The active pocket of MAO-B can be divided into two distinct cavities. The first is a narrow hydrophobic entrance cavity, enclosed by specific amino acid residues such as Phe103, Trp119, Leu164, Leu167, Phe168, Leu171, Ile198, Ile199, Ile316, and Tyr326 (Figure 5). This cavity, spanning an area of 290 Å, is situated near the lower portion of the protein and serves as a pathway for substrates or inhibitors to enter the catalytic pocket. The second cavity is referred to as the substrate cavity, running parallel to the FAD cofactor’s isoalloxazine ring. It has a flat, elongated shape with a volume of 420 Å and is lined with various aliphatic and aromatic amino acid residues, including Tyr60, Cys172, Gln206, Phe343, Tyr398, and Tyr435. These residues create an extremely hydrophobic environment that is suitable for binding inhibitors or substrates, and they are crucial for the correct alignment and stability of the protein–ligand complex. Two tyrosine residues, Tyr398 and Tyr435, positioned perpendicularly to the isoalloxazine ring of FAD, form an aromatic cage that plays a pivotal role in the catalysis of oxidative deamination [49,50,51].

For docking analysis, PDB ID: 2V5Z was used to check the potential of the derivatives against the MAO-B enzyme. This protein was chosen due to its human origin with a co-crystallized ligand. Many computational studies were conducted using the human MAO-B co-crystal structure, specifically focusing on chain B in the 2V5Z structure. This chain was chosen as it presented a more fitting model based on density considerations. Notably, the researchers observed that the crystallographic structure with the PDB code 2V5Z emerged as the most prominent and suitable for their investigation [52,53].

**Figure 6:**
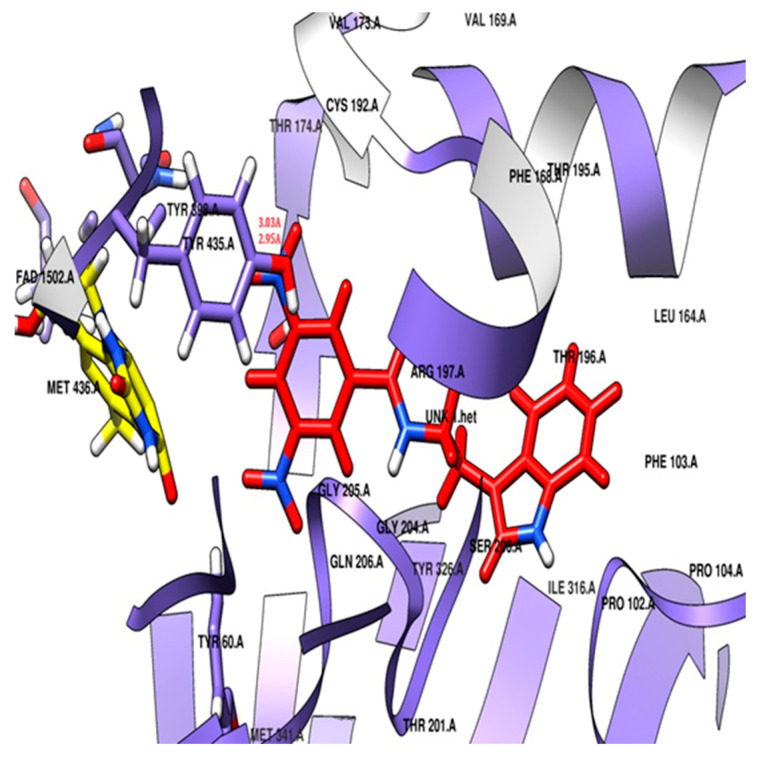
Potential docking position of **SR42** (red stick model) with 2V5Z protein.

The docking study revealed that the nitro-substituted ring of **SR42** is nestled in the aromatic cage (Tyr435, Tyr398, and FAD), where the two hydrogen bonds’ interactions with the one nitro with Tyr435 further aided its accommodation in the enzyme-substrate pocket, whereas the linker along with amine ensured further stability by interacting with Tyr60, Phe343, and Cys172. The indole ring of the molecule extended into the entrance pocket, generating a stable orientation facilitated by Phe103, Trp119, Leu164, Leu167, Phe168, Leu171, Ile198, Ile199, Ile316, Tyr326, and Leu328 (Figure 6). Pargyline, a selective MAO-B inhibitor, also adopted a docking pose that interacted with both the entrance pocket and the substrate binding pocket. These interactions were facilitated by a set of hydrophobic amino acid residues, including Tyr60, Phe168, Leu171, Cys172, Ile198, Ile199, Tyr326, Phe343, Tyr398, and Tyr435. However, pargyline is primarily engaged in hydrophobic interactions with the FAD cofactor. There was no hydrogen bond with the pargyline, and a decreased number of hydrophobic interactions were found in the pargyline as compared to **SR42**.

### 2.4. Molecular Docking of Cyclooxygenase-2 Enzyme

**Figure 7:**
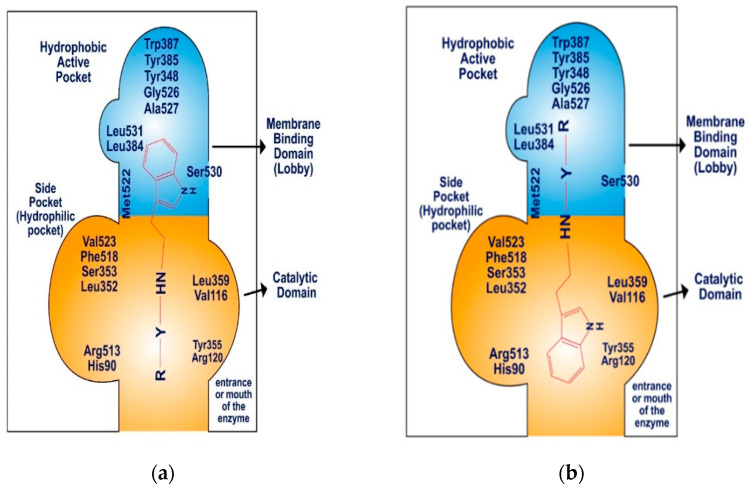
Schematic diagram: (**a**) binding pattern of SR22 within COX-2 enzyme, (**b**) binding pattern of SR24 and SR42 within COX-2 enzyme.

The COX-2 enzyme comprises three distinct components: a membrane-binding domain, also referred to as the “lobby”, a catalytic domain, and an epidermal growth factor domain. The active site of COX-2 features a hydrophobic channel that spans from the membrane-binding domain to the base of the catalytic site. The catalytic and epidermal growth factor domains create a dimerization point, aligning the two binding domains on the same plane, approximately 25 Å apart (Figure 7). The membrane-binding domain, which forms the hydrophobic pocket within the COX-2 active site, is lined with aromatic and hydrophobic amino acid residues, including Tyr348, Val349, Phe381, Leu384, Tyr385, Trp387, Met522, Gly526, Ala527, Ser530, and Leu531. The catalytic domain, specifically Val116 and Leu359, plays a significant role in binding nonsteroidal anti-inflammatory drugs (NSAIDs) [54,55,56].

Molecular docking against COX-2 (PDB ID: 4COX) was conducted for **SR22**, **SR24**, and **SR42**, showing promising inhibitory potential. The selection of 4COX was primarily due to the presence of indomethacin as a native ligand within the protein. Indomethacin shares the indole nucleus of synthesized tryptamine derivatives. Additionally, the choice of this protein is substantiated by its frequent appearance in recent publications, signifying its relevance and significance in ongoing research efforts [57,58].

Celecoxib, a standard compound with a docking score of −7.3 kcal/mol, interacted with both the catalytic and membrane-binding areas, involving hydrophobic amino acid residues (e.g., His90, Val116, Tyr348, Val349, Leu352, Tyr355, Leu359, Leu384, Tyr385, Trp387, Phe518, Met522, Val523, Gly526, Ala527) within the hydrophobic pocket, hydrophilic side pocket, and the catalytic domain. Celecoxib’s amine and sulfide groups formed hydrogen bonds with His90, Gln192, and Ser353 in the hydrophilic side pocket.

**Figure 8:**
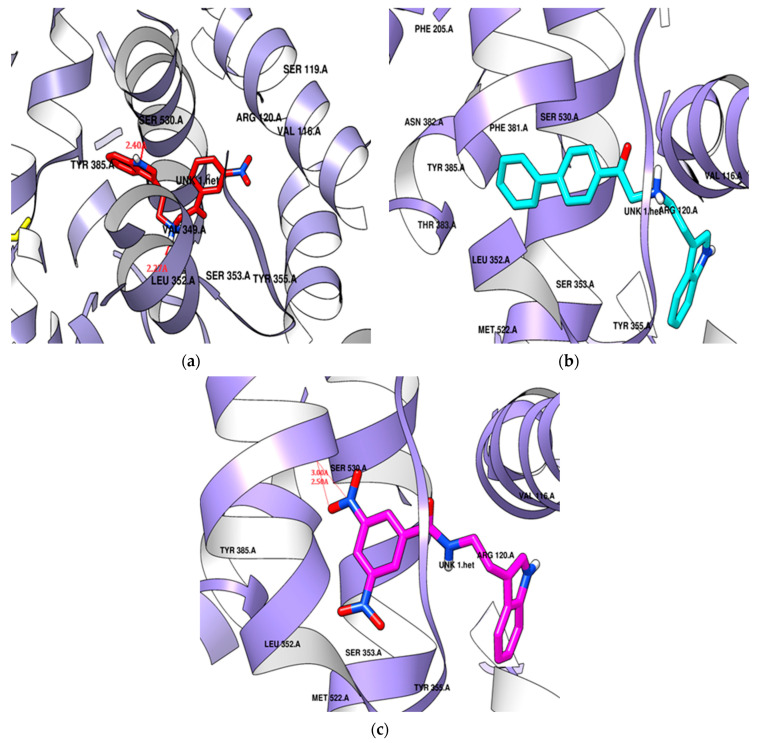
Potential docking position of (**a**) SR22 presented in a red color stick model, (**b**) SR24 presented in a cyan color stick model, (**c**) SR42 presented in a pink color stick model with 4COX protein.

The *para*-phenyl derivative **SR24**, with its substituted aromatic ring, exhibited a higher docking score (−9.6 kcal/mol) than the standard. **SR24** aligns its alkylamine moiety horizontally within the membrane-binding domain, interacting with ten amino acid residues (Leu352, Gly354, Phe381, Leu384, Tyr385, Trp387, Met522, Gly526, Ala527, Leu531), and the indole ring was positioned vertically within an inner hydrophilic pocket (His90, Val523, Phe518) and further stabilized itself by interacting with the catalytic domain through Val116 and Tyr355. The indole ring was twisted in the catalytic domain, and the two-benzene rings arranged in the series increased the length and size of the molecule oriented deeply into the hydrophobic pocket and showed good in vitro COX-2 inhibition activity (Figure 8b).

**SR42**, a di-nitro benzoyl tryptamine derivative, demonstrated a superior docking score (−9.1 kcal/mol) compared to the standard. The molecule entered the enzyme’s active site horizontally, with its indole ring setting at the enzyme’s mouth and the remaining part extended to the catalytic domain, where it interacted with Leu359 and Val116. **SR42** also interacted with the hydrophilic side pocket via His90, Phe518, and Val523. Ten hydrophobic hotspot residues (Tyr348, Val349, Leu352, Tyr355, Tyr385, Trp387, Met522, Gly526, Ala527, Leu531) engaged with an alkylamine, forming interactions within the enzyme’s lobby. Both the ends of the molecule were twisted in the active pocket, facing each other. The one nitro group of **SR42** formed two hydrogen bonds with Ser530 in the membrane-binding domain, which might play a role in showing good in vitro activity (Figure 8c).

**SR22**, a *meta*-nitro phenacyl derivative, presented an energy score (−8.3 kcal/mol) better than celecoxib. This molecule was situated within a hotspot site by Tyr355, blocked the enzyme’s mouth, and was then captured within a side hydrophilic pocket, interacting with His90 and Val523 through its *meta*-nitro phenacyl group. The indole ring engaged with seven hydrophobic amino acid residues (Tyr348, Val349, Leu352, Leu384, Met522, Gly526, Ala527) surrounding the membrane-binding pocket. **SR22** also interacted within the catalytic domain via Val116 and Leu359, forming two hydrogen bonds: one with Ser530 by the nitrogen of the indole ring and another with Leu352 by the alkylamine in hydrophobic and hydrophilic pockets. The indole ring occupied the hydrophobic cavity completely, and the involvement of hydrogen bonds both in the hydrophobic and hydrophilic area gave good in vitro COX-2 inhibition (Figure 8a).

## 3. Experimental

### 3.1. Biological Evaluation

#### 3.1.1. Acetylcholinesterase Inhibition

The AChE inhibitory potential of the synthesized tryptamine derivatives was investigated using a modified Ellman’s method. In this enzymatic assay, thiocholine, the product of enzymatic hydrolysis, lacks a substantial chromophore for UV detection. Therefore, Ellman’s reagent (DTNB) was employed as the chromogenic chemical. Ellman’s procedure for the in vitro acetylcholinesterase inhibition assay is based on a two-step reaction involving the chemical components DTNB (5,5-dithiobis(2-nitro) benzoic acid), a hydrolyzed substrate (thiocholine), and the enzyme AChE [59]. In the first step, the substrate acetylthiocholine iodide undergoes hydrolysis catalyzed by the AChE enzyme, resulting in the formation of thiocholine and acetic acid. In the second step, TNB (5-thio-2-nitrobenzoic acid) is generated by the reaction between Ellman’s reagent (DTNB) and thiocholine through an electron-shifting process to sulfur. This reaction produces TNB, which is characterized by its yellow color and can be quantitatively measured at a wavelength of 412 nm. Higher enzyme activity is reflected by an increase in absorbance at this wavelength. The formation of TNB is directly associated with the hydrolysis of acetylthiocholine iodide [60]. Each reaction was initiated by taking a 2 mL working solution, which consisted of 150 µL of the sample (either test compounds or positive control), 50 µL of 0.22 units/mL acetylcholinesterase enzyme from electric eel origin (AChE, E.C. 3.1.1.7, in lyophilized powder, ≥1000 unit), and sodium phosphate buffer (0.1 M, pH 7.4), making it up to a total of 2 mL. The working solution was then incubated at room temperature (37 °C) for 15 min, after which 1 mL of Ellman’s solution (200 µL of 75 mM ACTI and 500 µL of 10 mM DTNB in 15 mL sodium phosphate buffer) was added. The stock solutions of tryptamine analogs were prepared in dimethyl sulfoxide (DMSO) 100%. The reaction buffer was used to prepare different dilutions of the derivatives to obtain less than 2% (*v*/*v*) DMSO to avoid false positive results. All chemicals were purchased from Sigma-Aldrich, St. Louis, MO, USA and TCI Tokyo Chemical Industry Co., Ltd., Saitama, Japan. Following a rapid vortex of the solution, the chromophore formed from the hydrolysis of the ATCI substrate by the AChE enzyme was detected at 412 nm using a Shimadzu UV-1800 spectrophotometer. A negative control reaction was also performed under the same conditions but without the inclusion of samples. In this assay, the blank consisted of sodium phosphate buffer. Each experiment was conducted in triplicate.

The percentage of acetylcholinesterase inhibition activity for each tested dilution was calculated using the following equation:

$$Percent\;inhibition\;activity\left(\%\right)=\frac{Absorbance\;of\;control-Absorbance\;of\;test}{Absorbanec\;of\;control}\times 100$$

The results were then presented in a tabular format as the mean IC_50_ ± SD. The IC_50_ values, which represent the half-maximal inhibitory concentration, were determined by creating a graph that relates different concentrations (µM) to the percent inhibition (%) using the linear regression method provided by GraphPad Prism software 7 [61,62,63].

#### 3.1.2. Monoamine Oxidase Inhibition Assay

##### Extraction of MAO Enzyme from Synaptosomes of Rat Brain

Synaptosomes containing the MAO enzyme were isolated from the rat brain by the method of Hojas, with some modification [64]. Three Dawley rats of any sex with weight ranging from 200–250 gm were decapitated after administration of an anesthetic agent (mixture of propofol and xylocaine). Immediately, complete brains were removed from the bed of ice on a petri dish, and the brains were washed with ice-chilled PBS (phosphate-buffered saline) of pH 7.0, 0.1M and normal saline. Then, each brain, weighing approximately 1 g, was suspended into 10 mL of ice-chilled 3M sucrose pH 7.4 20% *w*/*v* accordingly and homogenized for 10 min using Daihan homogenizer HG-15A. Brain homogenate was transferred into a centrifuge tube and centrifuged at a speed of 7500× *g* rpm for 15 min at 4 °C by a Scan Speed 1730R Microcentrifuge from Labo Gene, Marlborough, MA, USA. The supernatant was preserved, and the solid brain mass debris present on the bottom was discarded. The supernatant was then diluted with ice-chilled distilled PBS as per need and centrifuged over 12,000× *g* rpm for 20 min at 4 °C through the mentioned microcentrifuge. Then, pellets containing a white solid mass at the bottom (synaptosomes containing the MAO enzyme) were stored, and each pallet was diluted with PBS buffer. Aliquoting of synaptosomes was carried out and stored at −70 °C till the assay was performed.

##### In Vitro Monoamine Oxidase Inhibition Assay

For the evaluation of the MAO-B inhibition potential of synthesized compounds, the Amplex^®^ Red Monoamine Oxidase Assay kit was purchased from Invitrogen Molecular Probes, Thermo Fisher Scientific, Waltham, MA, USA. This is a one-step fluorometric bioassay that principally detects the resorufin (7-Hydroxy-3H-phenoxazin-3-one), which is a fluorescent moiety formed in the presence of Horseradish Peroxidase (HRP) by the oxidation of nonfluorescent compound Amplex Red (AMR), chemically known as 10-acetyl-3,7-dihydroxyphenoxazine. The hydrogen peroxide (H_2_O_2_) was synthesized during the conversion of substrate to its aldehyde product by MAO enzyme action. H_2_O_2_ was used as an electron donor to form the fluorescent compound resorufin, which generally shows absorption at 571 nm and emission at 585 nm. The assay was performed according to the instruction of the manufacturer (http://tools.thermofisher.com/content/sfs/manuals/mp12214.pdf; accessed on 20 October 2020), with a few modifications. The Amplex Red reagent kit was used as compared to other H_2_O_2_ detection probes because of its stability and the stability of resorufin, which is a reaction product along with the longer wavelength spectra [39]. Extracted MAO-B enzyme from rat brain was diluted in sodium phosphate buffer of pH 7.4, 0.25 M. The substrate for MAO-B was benzylamine. Pargyline was used as a standard. All the stock solutions of inhibitors were prepared in dimethyl sulfoxide (DMSO) 100%. Different dilutions of inhibitors were prepared in reaction buffer to obtain less than 2% (*v*/*v*) DMSO for evading false positive results. The final reaction volume contained a two-fold lower concentration of enzyme and the inhibitors. Twenty millimeters H_2_O_2_ working solution was diluted in the reaction buffer to prepare a final concentration of 10 µM H_2_O_2_ and used as positive control, and the reaction buffer with no H_2_O_2_ was the negative control. The reaction was started by adding 100 µL of working solution in each well of a black, opaque, 96-well microplate holding the controls and samples. A total of 50 µL of the MAO enzyme solution was added into each well. The composition of the working solution was 400 µM Amplex Red reagent, reaction buffer, 2 U/mL HRP, and 2 mM substrate solution. The possible interference that can be produced by the fluorescence of the compounds generated within the reaction mixture because of non enzymatic inhibition was eradicated by the addition of samples with Amplex Red only in the reaction buffer. The fluorescence of the blank (control with no enzyme) was subtracted to correct the background fluorescence. All the reactions were incubated at 37 °C for 30 min. The fluorescence was measured with excitation at 560 nm and emission at 585nm by a VARIOSKAN LUX microplate reader by Thermo Scientific, US. All the readings were taken in triplicate. The results were displayed in a tabular format, presenting the mean IC_50_ values along with their standard deviations. The IC_50_ values were determined by generating a graph that relates different concentrations (µM) to the corresponding percent inhibition (%) values. The linear regression method provided by GraphPad Prism software 7 was employed to calculate these IC_50_ values [65,66].

#### 3.1.3. In Vitro Fluorometric COX-2 Inhibition Assay

The evaluation of synthesized molecules for COX-2 inhibition potential was conducted using a fluorometric COX-2 inhibitor screening kit, as detailed in the manufacturer’s manual (http://www.biovision.com/documentation/datasheets/K547.pdf; accessed on 12 March 2020). The fluorometric COX-2 inhibition assay kit provides a simple, fast, sensitive, and consistent method appropriate for carrying out high-throughput analysis for COX-2 inhibitors. The assay is based on the formation of prostaglandin G2 (PG2), which is an intermediate made from arachidonic acid under the action of the COX-2 enzyme. The reagents were prepared, and testing procedures were executed according to these recommended guidelines. The fluorescence of the samples was measured in an opaque 96-well plate at a temperature of 37 °C, precisely 5 min after the initiation of the experiment, utilizing a microplate reader from Thermo Scientific, USA. All compounds were tested at a concentration of 100 µM in a DMSO-1% solution. Celecoxib and indomethacin served as standard drugs for comparison. Dilutions were prepared in sodium phosphate buffer 0.1 M, pH 7.4. All the reagents were purchased from Sigma-Aldrich, St. Louis, MO, USA. The mean fluorescence values were calculated to determine the percentage of inhibition achieved by each compound. The fluorescence produced by the COX probe is directly proportional to the PG2, which is measured at extension/emission (Ex/Em = 535/587 nm) [67,68]. Each experiment was conducted in triplicate, and the percent inhibition was calculated using the following formula:

$$\;\%\;Inhibition=\frac{fluorescence\;of\;control-fluorescence\;of\;test\;compound}{fluorescence\;of\;control}\times 100$$

### 3.2. Molecular Docking

Crystal structures of AChE (PDB ID: 4EY7), hMAO-B (PDB ID: 2V5Z), and hCOX-2 (PDB ID: 4COX) were retrieved from the RCBS Protein Data Bank (https://www.rcsb.org/, accessed on 20 October 2020). Chain A of the enzyme was selected for the ease of docking and visualization. All heteroatoms and water molecules were deleted except for co-factors, which are involved in interactions. The UCSF Chimera 1.10.2 dock prep tool was used to minimize the energy of the protein, hydrogen was added in all polar residues, and Gasteiger-Hückel charges were added through AMBERff14SB. Target proteins were further translated to PDBQT format via PyRx0.9.2. Three-dimensional structures of the standard compounds were obtained in SDF format from the PubChem database (https://pubchem.ncbi.nlm.nih.gov/, accessed on 20 October 2020). Two-dimensional structures of the tryptamine analogs were drawn by ChemDraw Ultra 8.0 (Cambridge software 8) and saved in MOL file format. The 2D structures of analogs were converted into respective 3D structures by Chem3D Ultra8.0 and minimized by mmff94 force field of Open Babel in PyRx0.9.2. Ligands were converted in AutoDock ligand (PDBQT). The respective co-crystallized ligand of the target proteins was re-docked to validate the accuracy of docking calculations [19]. Thus, RMSD assured the reproducibility and valid selection of software for a particular selected target. Docking was executed by AutoDock Vina of PyRx0.9.2 (https://pyrx.sourceforge.io/, accessed on 20 October 2020) using default parameters, with the Lamarckian genetic algorithm as a scoring function. For docking against enzymes, the grid box was set around the receptor-binding pocket, respectively, using bound ligand and active site residues. Eight docked conformers of each ligand were generated, out of which the conformer with lowest binding energy (kcal/mol) and RMSD value between 0.5 and 2 was selected. The final docked structure of the ligand was saved in SDF format, and results were imported in CSV format. Hydrogen bond, hydrophobic, and aromatic interactions were explored through the docking study. Chimera 1.10.2 (https://www.cgl.ucsf.edu/chimera/, accessed on 20 October 2020) was used to visualize potential interactions of the ligands with the hotspot area of the receptor in 3D. Gimp 2 software (https://www.gimp.org/, accessed on 20 October 2020) was used for taking screenshots of protein–ligand interactions [69,70,71,72,73,74].

## 4. Conclusions and Future Perspective

There is a need to find more effective and safe drugs to stop the progression of Alzheimer’s disease, as only symptomatic therapies are available. Due to complex pathological cascades of AD, the development of MTDLs has been considered as an alternative to the currently available single-target drug and combination drug therapy. Current FDA-approved therapeutic agents for AD like donepezil, rivastigmine, and galantamine are mainly based on cholinergic hypothesis. The present study is focused on the in vitro and in silico evaluation of synthesized tryptamine derivatives as therapeutic hybrid ligands against AD, keeping AChE as the key target in combination with MAO-B and COX-2 inhibitions. Among all compounds, **SR42**, **SR25**, and **SR10** exhibited significant inhibitory activity against AChE, whereas only SR42 demonstrated noticeable inhibition against MAO-B as compared to all other compounds. Against COX-2 enzymes, **SR42**, **SR25**, and **SR10** exhibited good activity, although their activity did not surpass that of standard inhibitors, but these compounds succeeded in producing more than 70% inhibition of the enzyme. The docking picture revealed binding modes and interactions where these compounds fitted well into the enzyme active pocket. In view of the structure–activity relationship, carbonyl as a linker and terminal aromatic ring with *meta*-disubstituted electron-withdrawing groups induced comparatively better multi-target activity in **SR42**. Although these findings highlighted the potential of **SR42** against the selected targets, structural modifications are needed for better efficacy as well as a better safety profile. Advanced in silico studies like molecular dynamics simulations are necessary to obtain the kinetic information for the protein–drug interactions, and in-depth in vitro enzyme assays are needed for the enzyme selectivity and reversibility.

Presently, we are working on different pharmacophores including tryptamine as MTDLs for AD through AChE as the main target in combination with COX-2, MAO-B, and β-amyloid aggregation inhibition along with antioxidant activity. Future findings will provide a strong foundation to explore the tryptamine nucleus as a valuable avenue for designing and developing lead compounds with enhanced therapeutic potential to serve as multifactorial therapeutic agents for neurodegenerative diseases.

## Figures and Tables

**Table 1:** In vitro acetylcholinesterase inhibition, MAO-B inhibition, and COX-2 inhibition activities.

S.no.	Compound Code	AChE ^b^ (IC_50_^a^ ± SD (µM)	MAO-B ^c^ (IC_50_^a^ ± SD (µM)	COX-2 ^d^ (%Inhibition ^a^ ± SD)
1	**SR08**	4.36 ± 0.12	244.20 ± 1.00	72.33 ± 1.67
2	**SR10**	1.00 ± 0.08	216.10 ± 0.29	72.90 ± 2.42
3	**SR13**	9.68 ± 0.16	50.64 ± 0.64	73.83 ± 2.37
4	**SR14**	68.71 ± 0.24	112.20 ± 0.29	65.26 ± 2.65
5	**SR17**	8.23 ± 0.12	150.10 ± 0.26	72.83 ± 2.54
6	**SR20**	24.97 ± 0.05	222.13 ± 0.32	68.66 ± 3.86
7	**SR21**	87.73 ± 0.16	142.13 ± 0.32	34.43 ± 3.34
8	**SR22**	54.88 ± 0.19	56.46 ± 0.41	84.08 ± 4.53
9	**SR23**	7.33 ± 0.12	95.10 ± 0.43	71.40 ± 3.11
10	**SR24**	6.70 ± 0.08	133.20 ± 0.46	79.30 ± 2.02
11	**SR25**	0.17 ± 0.02	85.10 ± 0.26	72.43 ± 2.36
12	**SR42**	0.70 ± 0.21	43.21 ± 0.46	75.16 ± 2.30
13	Tryptamine	88.26 ± 0.24	288.13 ± 0.32	57.33 ± 4.96
14	Donepezil	1.96 ± 0.41	--	--
15	Galantamine	23.37 ± 0.26	--	--
16	Pargyline	--	23.37 ± 0.32	--
17	Celecoxib	--	--	87.86 ± 0.63
18	Indomethacin	--	--	87.03 ± 0.57

^a^ Each value is the mean of three independent measurements. ^b^ AChE from electric eel was used. ^c^ MAO-B was extracted from the rat brain. ^d^ Human recombinant COX-2 was used.

**Table 2:** Docking scores and interacting residues of standards and ligands against AChE, MAO-B, and COX-2 enzymes (PDB ID: 4EY7, 2V5Z, and 4COX).

PDB ID: 4EY7 (AChE)				
S. No.	Ligand Code	Docking Score (kcal/mol)	Hydrogen Bond Interaction (3Å)	Hydrophobic Interacting Amino Acids (5Å)
1.	**SR42**	−10.4	Gly121, Tyr124, His447	Tyr72, Trp86, Gly120, Gly121, Tyr124, Trp286, Phe295, Phe297, Tyr337, Phe338, Tyr341, His447, Gly448
2.	**SR10**	−8.7	Tyr337	Trp86, Gly120, Gly121, Tyr124, Phe295, Phe297, Tyr337, Phe338, Tyr341, His447, Gly448
3.	**SR25**	−9.7	Tyr124	Tyr72, Trp86, Gly120, Gly121, Tyr124, Tyr133, Trp286, Phe295, Phe297, Tyr337, Phe338, Tyr341, His447, Gly448
4.	**Donepezil**	−11.2	Tyr124, Phe295	Tyr72, Trp86, Gly120, Gly121, Tyr124, Trp286, Phe295, Phe297, Tyr337, Phe338, Tyr341, His447, Gly448
**PDB ID:2V5Z (MAO-B)**				
S. No.	Ligand code	Docking score (kcal/mol)	Hydrogen bond interaction (3Å)	Hydrophobic interacting amino acids (5Å)
1.	**SR42**	−9	Tyr435	Tyr60, Phe103, Leu167, Phe168, Leu171, Cys172, Ile198, Ile199, Thr201, Ile316, Tyr326, Tyr398, Tyr435, FAD
2.	**Pargyline**	−7.4	---	Tyr60, Phe168, Leu171, Cys172, Ile198, Ile199, Thr201, Tyr326, Phe343, Tyr398, Tyr435, FAD
**PDB ID: 4COX (COX-2)**				
S. No.	Ligand code	Docking score (kcal/mol)	Hydrogen bond interaction (3Å)	Hydrophobic interacting amino acids (5Å)
1.	**SR22**	−8.3	Leu352, Ser530	His90, Val116, Tyr348, Val349, Leu352, Leu359, Leu384, Tyr355, Met522, Val523, Gly526, Ala527, Leu531
2.	**SR24**	−9.6	---	His90, Val116, Tyr348, Val349, Leu352, Leu359, Leu384, Tyr355, Met522, Val523, Gly526, Ala527, Leu531
3.	**SR42**	−9.1	Ser530	His90, Val116, Tyr348, Val349, Leu352, Tyr355, Leu359, Tyr385, Trp387, Phe518, Met522, Val523, Gly526, Ala527, Leu531
4.	**Celecoxib**	−7.3	His90, Gln192, Ser353	His90, Val116, Tyr348, Val349, Leu352, Tyr355, Leu359, Phe518, Met522, Val523, Gly526, Ala527, Leu531

## Data Availability

Data are contained within the article.
